# Public expenditure on hospitalizations for COVID-19 treatment in 2020, in Brazil

**DOI:** 10.11606/s1518-8787.2021055003666

**Published:** 2021-08-04

**Authors:** Hebert Luan Pereira Campos dos Santos, Fernanda Beatriz Melo Maciel, Geovani Moreno Santos, Poliana Cardoso Martins, Nília Maria de Brito Lima Prado

**Affiliations:** I Universidade Federal da Bahia Instituto Multidisciplinar em Saúde Vitória da ConquistaBA Brasil Universidade Federal da Bahia. Instituto Multidisciplinar em Saúde - Campus Anísio Teixeira. Vitória da Conquista, BA, Brasil; II Universidade Federal da Bahia Instituto Multidisciplinar em Saúde Programa de Pós-Graduação em Saúde Coletiva Vitória da ConquistaBA Brasil Universidade Federal da Bahia. Instituto Multidisciplinar em Saúde - Campus Anísio Teixeira. Programa de Pós-Graduação em Saúde Coletiva. Vitória da Conquista, BA, Brasil

**Keywords:** Coronavirus Infections, Hospitalization, Hospital Costs, Public Expenditures on Health, Unified Health System

## Abstract

**OBJECTIVE:**

Describe the expenditure resulting from hospitalizations for clinical treatment of users diagnosed with COVID-19 in the Unified Health System (SUS) between February and December 2020.

**METHODS:**

This is a descriptive study based on data from the Hospital Information System about government expenditure on hospitalizations for clinical treatment of users diagnosed with COVID-19 and causes included in the ICD-10 chapters. We obtained the number of hospitalizations, average length of stay, lethality rate, and total expenditure considering hospital services, professional services and average expenditure per hospitalization.

**RESULTS:**

In the period evaluated, SUS registered 462,149 hospitalizations, 4.9% of them for COVID-19 treatment. Total expenditure exceeded R$ 2.2 billion, with 85% allocated to hospital services and 15% to professional services. Expenditure for treating COVID-19 was distributed differently between the country’s regions. The Southeast region had the highest number of hospitalizations, highest total amount spent, highest average length of stay in days, and highest lethality rate; the South region, in turn, recorded the highest percentage of spending on non-profit hospitals (58%) and corporate hospitals (15%).

**CONCLUSIONS:**

Hospitalizations for clinical treatment of coronavirus infection were more costly compared to those for treatment of acute respiratory failure and pneumonia or influenza. Our results show the disparities in hospitalization expenditure for similar procedures between the regions of Brazil, underlining the vulnerability and the need for strategies to reduce the differences in access, use, and distribution of SUS resources, ensuring equanimity, and considering the unfair inequalities between the country’s regions.

## INTRODUCTION

SARS-CoV-2 infection has emerged as a major challenge for health systems. Since its outbreak in Wuhan, China in December 2019, the disease caused by the new coronavirus has totaled more than 161,513,458 confirmed cases and 3,352,109 deaths worldwide as of May 15, 2021^1^ . Clinically, the infection can manifest in three distinct ways, which vary according to age group and associated clinical conditions: asymptomatic carrier, individual with acute respiratory disease (ARD) or patients with pneumonia at different degrees of severity^2^ .

COVID-19 is an acute respiratory infections (ARI), a group of clinical syndromes whose most common infectious agents are respiratory viruses, such as syncytial, or bacteria such as *Streptococcus pneumoniae* and *Haemophilus influenzae* ^3^ . The severity of the disease will depend on the infectious agent, the environment and host factors. Thus, according to the World Health Organization (WHO), ARI are among the infectious diseases with the highest morbidity and mortality rates worldwide^4^ . Data show that these infections are responsible for more than 4 million deaths per year, representing a major cause of death in developing countries^5^ .

In Brazil, in periods prior to the pandemic, such infections accounted for nearly one fifth of hospitalizations in the Unified Health System (SUS)^6^ . COVID-19 alone, as of May 15, 2021, recorded more than 432,000 deaths and 15,519,525 infected, but its impacts on the health sector and the national economy began to be noticed already in 2020^7 , 8^ . Importantly, Brazil is among the few countries in the Americas to have a universal and free healthcare system, on which about 75% of the population depends exclusively, and which is recognized as one of the country’s most important public policies^9^ .

SUS is cited as an example of a successful healthcare system in Latin America, mainly for guaranteeing health as a right of all Brazilian citizens^9^ . On the other hand, the underfunding challenges have put the guarantee of this right at risk^10^ . In the context of the pandemic, the role played by SUS is significant, especially in hospital care. COVID-19 has resulted in additionally high hospitalization rates^11^ , and besides drawing attention to the added pressure on the health system, it is worth observing and analyzing SUS expenditure and how it is distributed throughout the territory.

In the scenario of the COVID-19 pandemic, developing analyses to estimate the direct medical-hospital costs required – including examinations, procedures, drugs, human resources, appointments, hospitalizations, rehabilitation and^12^ –, becomes indispensable to support decision-making, in terms of meeting the demands on services and development of operational strategies by national health systems^13^ .

Searches conducted on the Virtual Health Library (VHL), Scientific Electronic Library Online (SciELO) and PubMed, using the Descriptors in Health Sciences (DEcS/Mesh) in English and Portuguese, “hospital costs,” “health expenditures,” “public expenditures on health,” “public expenditures” and “public expenditures on private services” associated with the terms “COVID-19” and “Brazil,” using the Boolean operator AND, returned no prior study, of national scope, describing SUS expenditure on hospitalizations for clinical treatment of confirmed COVID-19 cases. Research on these expenditures, therefore, is minor in the literature, although addressing such a topic is extremely relevant to understand its toll on the public health system.

This study, thus, seeks to analyze the percentage of public expenditures on SUS hospitalizations for clinical treatment of COVID-19 patients in Brazil, between February and December 2020. To this end, we described and analyzed public expenditures on hospitalizations and how they are distributed according to the population characteristics. Our findings may contribute to adopting measures capable of avoiding complications and, consequently, the collapse of the healthcare system in a pandemic scenario.

## METHODS

This is a descriptive study based on secondary data about government expenditures on hospitalizations for clinical treatment of users diagnosed with COVID-19 in Brazil, from February to December 2020.

Data on hospitalizations and their respective costs were collected from the Unified Health System’s Hospital Information System (SIH-SUS), available at the SUS Department of Information Technology (Datasus)^14^ website, through consolidated information on inpatient hospital authorizations (IHA), classified by country region.

The selected hospitalizations had as their main procedure code 03.03.01.022-3 (treatment of coronavirus infection) – which corresponds to the actions necessary to clinically treat hospitalized users diagnosed with coronavirus infection, in accordance with Ordinance No. 245, of March 24, 2020, and the Technical Guidelines for SIH-SUS operationalization during the coronavirus state of public health emergency, updated by the Ministry of Health on August 13, 2020^15^ –, in hospital units linked to SUS (public and private associated).

We also sought to compare the expenditures on hospitalizations for treatment of patients diagnosed with COVID-19, expenditures on hospitalizations for all causes considering the ICD-10 chapters, and expenditures on treatment of major respiratory system infections, such as pneumonia, influenza and acute upper and lower tract infections. For the latter, we selected procedures with code: 03.03.14.015-1 – treatment of pneumonia or influenza (flu), 03.03.14.010-0 – treatment of acute upper tract infections, and 03.03.14.014-3 – treatment of other acute lower tract infections^16^ .

Our study excluded data on treatments without scientific evidence for coronavirus infection. Hospitalization for treatment comprises actions and procedures necessary to stabilize and prevent the worsening of the user’s clinical picture. Such interventions, however, can vary substantially depending on the clinical picture of each user (considering associated comorbidities and degree of lung involvement), between the clinical protocols adopted by each state and municipality, and between the clinical practices chosen.

The chosen time frame comprises the months of February to December 2020 for hospitalizations for clinical treatment of users diagnosed with coronavirus. Such time frame is justified because the first case of SARS-CoV-2 infection in Brazil was confirmed on February 26, 2020; while December was the last month with data available in the SIH-SUS.

The variables of interest taken from the SIH-SUS comprised: number of hospitalizations, total cost of hospitalizations, cost of professional services, cost of hospital services, average cost of SUS hospitalization (by specialty or procedure, in a given geographical space, in the year considered), average length of stay (average total number of days for the IHA approved in the period) and lethality rate (ratio between the number of deaths and the number of approved IHA in the period, computed as hospitalizations, multiplied by 100)^17^ . All expenditures were calculated in reais, and the average length of stay was counted in days.

We also sought to identify the total amount paid by legal sphere, that is, the distribution of total government expenditures according to legal sphere, considering the categories of the National Registry of Health Establishments^18^ : “public hospitals,” “corporate hospitals,” and “non-profit hospitals.” The first group consists of those whose legal sphere is “federal public administration,” “state or Federal District,” “municipal” and “public administration – others.” Corporate are those whose legal sphere is “public company or mixed-capital company” and “other business entities.” Non-profit hospitals, in turn, are those registered in the legal sphere “non-profit entities”^18^ .

The number, proportion, ratio and amounts paid by hospitalizations, obtained from the SIH-SUS, were tabulated using the Microsoft Excel program and calculated by descriptive statistical analysis.

The analysis was performed on secondary data, of open access, without the possibility of individual identification of the information. Thus, according to the recommendations of the National Health Council (CNS) Resolution No. 466 of December 12, 2012, the ethical principles of research involving human beings were respected, and the research ethics committee approval waived.

## RESULTS

Between February and December 2020, the SIH-SUS registered 462,149 hospitalizations, having as their main procedure the treatment of users with coronavirus, corresponding to a total expenditure of R$ 2,248,011,968.40. Importantly, SUS spent 85% of this amount on hospital services and 15% on professional services. Among all regions of the country, the Southeast accounted for the largest share of expenditures, representing about 45% of the total. The South and North regions presented, respectively, the highest and lowest average cost per hospitalization ( Table 1 ).

**Table 1:** Expenditures (in reais) of hospitalizations for clinical treatment of coronavirus infectiona according to region, Brazil, February – December 2020.

Region	Total amount of expenditure^b^ (R$)	Cost of hospital services^c^ (R$)	Cost of professional services^d^ (R$)	Average cost per hospital hospitalization^e^ (R$)
North	133,723,986.43	111,708,340.36	22,015,646.07	3,157.96
Northeast	550,997,791.12	465,375,038.13	85,622,752.99	4,489.91
Southeast	1,010,529,436.45	857,747,748.55	152,776,809.87	5,154.34
South	381,184,490.20	324,887,362.21	56,297,127.99	6,165.64
Midwest	171,576,264.24	145,114,948.61	26,460,690.51	4,376.16

**Total**	**2,248,011,968.44**	**1,904,833,437.86**	**343,173,027.43**	**4,864.26**

Source: Own elaboration, based on data from SIH-SUS.

^a^ Hospitalizations of patients with COVID-19 registered with procedure code 03.03.01.022-3, compliant with Ordinance No. 245 of March 24, 2020 and Technical Guidelines for SIH-SUS operationalization during the coronavirus state of public health emergency published on August 13, 2020 by the Ministry of Health.

^b^ Amount for IHA approved in the period. This amount does not necessarily correspond to the sum allocated to the establishment – depending on the situation of the units, they receive budgetary resources or there may be withholdings and incentive payments, not presented here. This amount should, therefore, be considered as the approved production sum.

^c^ Cost of hospital services for IHA approved in the period.

^d^ Cost of professional services for IHA approved in the period.

^e^ Average cost per hospitalization in the Unified Health System (SUS), by specialty, in a given geographical space, in the period considered.

When considering all hospitalizations taken place in Brazil under SUS, in the analyzed period, 4.9% had as main procedure the treatment of COVID-19, corresponding to 15% of the expenditures with all hospitalizations in the country. By comparing the average length of stay (in days) between all hospitalizations and hospitalizations for coronavirus treatment, we found that the latter lasted longer ( Table 2 ).

**Table 2:** Expenditures (in reais) and average length of stay (in days) of hospitalizations for ICD-10 chapters versus hospitalizations for clinical treatment of coronavirus infection according to region, Brazil, February – December 2020.

Region	Hospitalizations for ICD-10 chapters^a^				Hospitalizations for treatment of coronavirus infection^b^					
	
	Hospitalizations	Total amount of expenditure^c^ (R$)	Average cost per hospital hospitalization^d^	Average length of stay (in days)^f^	Hospitalizations	%^e^ total hospitalizations	Total amount of expenditure^c^ (R$)	%^e^ total amount of expenditure on hospitalizations	Average cost per hospital hospitalization^d^	Average length of stay (in days)^f^
North	758,143	819,819,970.59	1,081.35	4.7	42,345	5.6	133,723,986.4	16.3	3,157.96	7.4
Northeast	2,461,730	3,462,075,135.95	1,406.36	5.3	122,719	5.0	550,997,791.1	15.9	4,489.91	7.8
Southeast	3,772,966	6,511,672,543.21	1,725.88	5.9	196,054	5.3	1,010,529,436	15.5	5,154.34	8.6
South	1,646,796	3,055,201,666.37	1,855.24	5.2	61,824	3.7	381,184,490.2	12.5	6,165.64	8.5
Midwest	746,075	1,064,013,109.82	1,426.15	5.1	39,207	5.2	171,576,264.2	16.1	4,376.16	7.4

**Total**	**9,385,710**	**14,912,782,425.94**	**1,588.88**	**5.4**	**462,149**	**4.9**	**2,248,011,968.44**	**15**	**4,864.26**	**8.2**

Source: Own elaboration, based on data from SIH-SUS.

^a^ All hospitalizations taken place under SUS, considering all ICD-10 chapters.

^b^ All hospitalizations taken place under SUS with procedure code 245, compliant with Ordinance No. 24 of March 2020, 13 and Technical Guidelines for SIH-SUS operationalization during the coronavirus state of public health emergency published on August 2020, 2020 by the Ministry of Health.

^c^ Amount for the IHAs approved in the period. This amount does not necessarily correspond to the sum allocated to the establishment – depending on the situation of the units, they receive budgetary resources or there may be withholdings and incentive payments, not presented here. This amount should, therefore, be considered as the approved production sum.

^d^ Average cost per hospitalization in the Unified Health System (SUS), by specialty, in a given geographical space, in the period considered.

^e^ Percentages calculated based on the cost of hospitalizations and total expenditures by region for all ICD-10 chapters.

^f^ Average length of stay for approved IHA, computed as hospitalizations, in the period.

**Figure 1:**
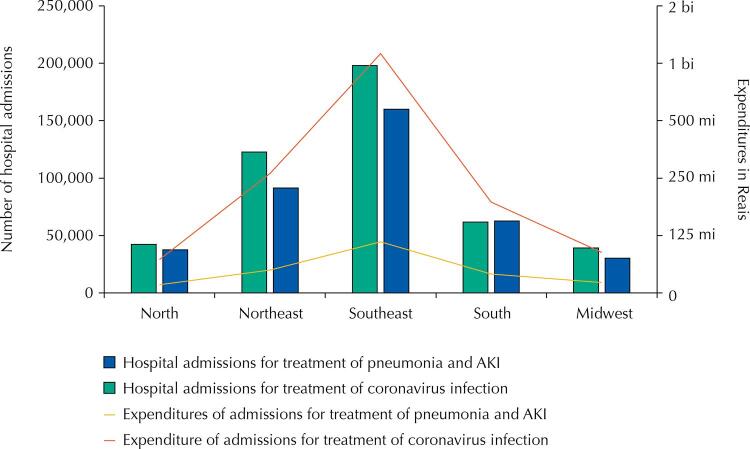
Hospitalizations and expenditures for treatment of coronavirus infectiona and for treatment of acute upper and lower tract infections, pneumonia or influenzab by region, Brazil, February – December 2020.

^a^ Procedure with code 03.03.01.022-3 (treatment of coronavirus infection) – which corresponds to the actions necessary to clinically treat hospitalized patients diagnosed with COVID-19.

^b^ Procedures with codes 03.03.14.015-1 – treatment of pneumonia or influenza (flu), 03.03.14.010-0 – treatment of acute upper tract infections, and 03.03.14.014-3 – treatment of other acute lower tract infections.

When comparing expenditures and the hospitalizations whose main procedure was treatment of acute upper and lower tract infections, pneumonia or influenza, with hospitalizations for coronavirus treatment, the higher expenses and number of hospitalizations found also refer to coronavirus infection ( Figure 1 ).

ARI: acute respiratory infections.

Regarding the expenditures associated with the average length of stay and lethality rate, comparing hospitalizations for COVID-19 treatment with hospitalizations for treatment of pneumonia or influenza, the Southeast region showed the highest number of hospitalizations, highest total amount spent, highest average length of stay in days, and higher lethality rate. The states of São Paulo and Minas Gerais had the highest expenditures for treatment of these conditions. The state of Roraima presented the longest length of stay for treatment, both of coronavirus infection and of pneumonia or influenza. The states of Paraíba and Rio de Janeiro had the highest lethality rates for coronavirus treatment and treatment of influenza or influenza, respectively ( Table 3 ).

**Table 3:** Expenditures (in reais), average length of stay (in days), and lethality rate (per 100 inpatients) of hospitalizations for clinical treatment of coronavirus infection versus hospitalizations for treatment of pneumonia or influenza (flu) according to region, Brazil, February – December 2020.

Regions	Hospitalizations for treatment of coronavirus infection^a^							Hospitalizations for treatment of pneumonia or influenza (flu)^b^						
	
	Hospitalizations		Total amount of expenditure^c^		Average cost per hospital hospitalization (in reais)^d^	Average length of stay (in days)^e^	Lethality rate^f^	Hospitalizations		Total amount of expenditure^c^		Average cost per hospital hospitalization (in reais)^d^	Average length of stay (in reais)^e^	Lethality rate^f^
			
	(n)	(%)	(R$)	(%)				(n)	(%)	(R$)	(%)			
**North**	**42,345**	**9**	**133,723,986,4**	**6**	**3,157.96**	**7.4**	**20.64**	**34,192**	**10**	**34,865,889.09**	**8**	**1,019.71**	**6.0**	**7.79**
Rondônia	4,550	1	18,287,032.7	1	4,019.13	6.2	15.52	2,969	1	4,256,679.93	1	1,433.71	6.4	9.16
Acre	1,566	0	5,902,502.86	0	3,769.16	9.2	22.03	1,059	0	1,426,106	0	1,346.65	8.0	16.9
Amazonas	12,276	3	38,513,890.99	2	3,137.33	8.0	23.37	5,568	2	5,489,313.14	1	985.87	6.2	10.63
Roraima	1,699	0	6,162,828.14	0	3,627.33	9.7	25.13	1,686	0	2,066,920.29	0	1,225.93	9.4	10.74
Pará	18,509	4	54,212,642.19	2	2,928.99	7.2	19.12	19,326	6	18,065,351.26	4	934.77	5.3	6.12
Amapá	2,418	1	4,898,168.88	0	2,025.71	7.2	24.69	1,562	0	1,313,542.34	0	840.94	7.5	5.76
Tocantins	1,327	0	5,746,920.67	0	4,330.76	5.7	19.29	2,022	1	2,247,976.13	0	1,111.76	6.8	8.31
**Northeast**	**122,719**	**27**	**550,997791.1**	**25**	**4,489.91**	**7.8**	**21.84**	**83,455**	**24**	**93,769,264.42**	**21**	**1,123.59**	**6.2**	**11.16**
Maranhão	18,685	4	55,102,185.73	2	2,949.01	6.9	19.19	14,013	4	13,795,977.17	3	984,51	5.2	6.93
Piauí	9,868	2	46,716,983.14	2	4,734.19	7.4	17.73	9,005	3	7,060,563.32	2	784,07	4.9	6.36
Ceará	23,122	5	95,454,894.14	4	4,128.31	7.7	22.04	16,054	5	17,545,693.07	4	1,092.92	7.0	13.98
Rio G. do Norte	6,464	1	32,079,037.42	1	4,962.72	7.2	24.09	3,774	1	4,317,075.87	1	1,143.9	7.2	12.75
Paraíba	6,136	1	29,309,295.2	1	4,776.61	8.4	31.83	5,757	2	6,396,642.12	1	1,111.11	6.7	14.33
Pernambuco	29,866	6	133,019,014.1	6	4,453.86	8.1	22.34	9,632	3	1,607,5631.99	4	1,668.98	7.6	12.52
Alagoas	5,338	1	239,012,90.05	1	4,477.57	8.6	25.42	4,548	1	6,343,639.79	1	1,394.82	6.3	10.64
Sergipe	4,080	1	19,007,855.07	1	4,658.79	9.2	22.97	2,194	1	2,708,212.73	1	1,234.37	8.1	19.74
Bahia	19,160	4	116,407,236.3	5	6,075.53	8.2	20.34	18,478	5	19,525,828.36	4	1,056.71	5.7	11.35
**Southeast**	**196,054**	**42**	**1,010,529,436**	**45**	**5,154.34**	**8.6**	**22.61**	**145,005**	**42**	**206,452,552.1**	**45**	**1,423.76**	**7.2**	**16.57**
Minas Gerais	34,553	7	196,447,903.6	9	5,685.41	8.2	19.58	41,118	12	596,589,07.46	13	1,450.92	6.8	13.05
Espírito Santo	10,839	2	68,060,298.71	3	6,279.2	8.7	21.82	6,671	2	9,073,085.62	2	1,360.08	6.6	10.9
Rio de Janeiro	38,865	8	139,621,866.1	6	3,592.48	8.3	27.9	22,279	6	30,502,415.18	7	1,369.11	8.2	22.77
São Paulo	111,797	24	606,399,368	27	5,424.11	8.8	21.78	74,937	22	10,721,8143.8	23	1,430.78	7.1	17.16
**South**	**61,824**	**13**	**381,184,490.2**	**17**	**6,165.64**	**8.5**	**19.25**	**58,222**	**17**	**77,888,586.08**	**17**	**1,337.79**	**6.0**	**13.74**
Paraná	22,702	5	131,733,713.6	6	5,802.74	7.9	19.27	22,580	6	30,765,544.92	7	1,362.51	5.2	10.7
Santa Catarina	14,270	3	95,031,703,47	4	6,659.54	8.2	18	14,135	4	19,970,729.02	4	1,412.86	5.6	13.56
Rio G. do Sul	24,852	5	154,419,073,2	7	6,213.55	9.1	19.95	21,507	6	27,152,312.14	6	1,262.49	7.2	17.04
**Midwest**	**39,207**	**8**	**171,576,264.2**	**8**	**4,376.16**	**7.4**	**18.37**	**27,445**	**8**	**43,362,970.17**	**10**	**1,580.00**	**6.5**	**11.96**
Mato G. do Sul	4,408	1	21,722,376.9	1	4,927.94	8.1	19.44	6,897	2	9,825,686.84	2	1,424.63	6.4	14.21
Mato Grosso	9,328	2	41,813,431.57	2	4,482.57	7.1	19.83	5,813	2	8,298,784.8	2	1,427.63	6.0	10.87
Goiás	16,100	3	70,691,166.46	3	4,390.76	6.5	18.8	10,795	3	18,489,557.09	4	1,712.79	6.1	12.24
Distrito Federal	9,371	2	37,349,289.31	2	3,985.62	8.9	15.68	3,940	1	6,748,941.44	1	1,712.93	8.4	8.88

**Total**	**462,149**	**100**	**2,248,011,968.44**	**100**	**4,864.26**	**8.2**	**21.42**	**348,319**	**100**	**456,339,261.8**	**100**	**1,310.12**	**6.6**	**13.57**

Source: Own elaboration, based on data from SIH-SUS.

^a^ Hospitalizations of patients with COVID-19 registered with procedure code 03.03.01.022-3, compliant with Ordinance No. 245 of March 24, 2020 and Technical Guidelines for SIH-SUS operationalization during the coronavirus state of public health emergency published on August 13, 2020 by the Ministry of Health.

^b^ Hospitalizations of patients registered on the Inpatient Hospital Authorization (IHA) with procedure code 03.03.14.015-1.

^c^ Amount for the IHAs approved in the period. This amount does not necessarily correspond to the sum allocated to the establishment – depending on the situation of the units, they receive budgetary resources or there may be withholdings and incentive payments, not presented here. This amount should, therefore, be considered as the approved production sum.

^d^ Average cost per hospitalization in the Unified Health System (SUS), by specialty, in a given geographical space, in the period considered.

^e^ Average length of stay for approved IHA, computed as hospitalizations, in the period.

^f^ Ratio between the number of deaths and the number of approved IHA, computed as hospitalizations, in the period, multiplied by 100.

**Figure 2:**
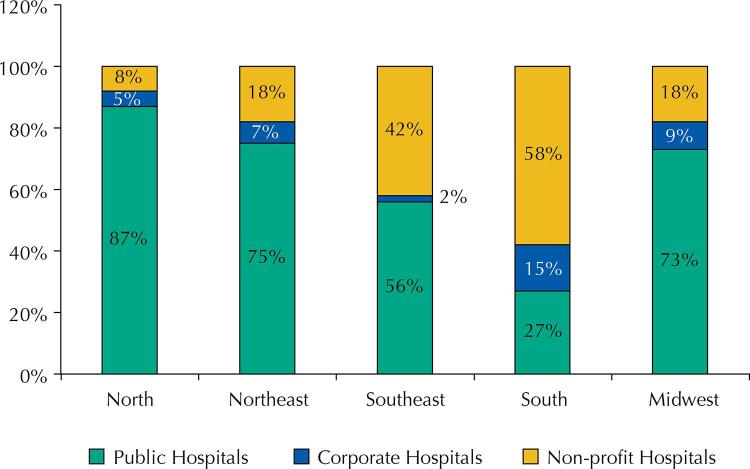
Distribution of expenditures on hospitalizations for clinical treatment of coronavirus infection between the legal spheres of SUS hospitals by region, Brazil, February – December 2020.

Source: Own elaboration, based on data from SIH-SUS.

As for the percentage of distribution of total hospitalization expenditure for COVID-19 treatment between the legal spheres of SUS hospitals, we found a higher percentage of expenditures with non-profit hospitals (58%) and corporate hospitals (15%) in the South region ( Figure 2 ).

## DISCUSSION

Our findings showed that the public expenditures of hospitalizations for COVID-19 treatment were distributed differently between the country’s regions.

Considering the volume of hospitalizations for clinical treatment of coronavirus-infected users in the period analyzed, it is evident the impact that such hospitalizations generate on the health system. Although the hospitalizations born by SUS do not account for all hospitalized cases, the progressive evolution of the patient’s clinical picture confers great care coverage to SIH-SUS for these hospitalizations.

Previous studies have also reported on the geographical variations of the average amount paid per hospitalization in Brazil, verified by the significant differences in regional expenditure, regardless of the COVID-19 pandemic^20^ . The average cost of hospitalization is higher for this treatment when compared to all causes of hospitalization and, more specifically, when compared to cases of treatment for pneumonia or influenza. Such fact indicates that hospitalizations for clinical treatment of COVID-19 are more expensive than those for other causes. Importantly, the variations between the average cost of hospitalization and the average length of stay result from socioeconomic, epidemiological and demographic factors, which determine the profile of hospital demand by SUS, as well as public healthcare policies^21^ .

This scenario is compounded by fiscal austerity measures and the strong restriction of revenues by the federal level – the main source of resources for service provision in the SUS. Although the transfer of specific federal financial resources so that states and municipalities can fund public actions and services related to combating COVID-19 was regulated, recent regulatory changes in financing, with no discretion in the allocation of resources by state and municipal entities, have intensified regional inequality in the supply of actions and services by the SUS, including outpatient and hospital services. With the approval, in 2016, of Constitutional Amendment No. 95, which established a spending ceiling for the Federal Government, SUS lost almost 22.5 billion reais between 2018 and 2020^22^ . In this respect, the pandemic imposed new challenges to the system, due to the community transmissibility characteristics of the disease^23^ and, according to the data presented here, the higher cost of hospitalizations.

Research shows that most COVID-19 patients evolve with few symptoms or even asymptomatic. According to the WHO^24^ , however, 14% of the identified cases develop severe disease, requiring hospitalization and oxygen therapy, 5% require hospitalization in Intensive Care Units (ICU), and most ICU patients require ventilatory support^24^ .

For data analysis and decision making, one must consider the heterogeneous distribution, between the country’s regions, of the population’s age structure and mobility, the percentage of people in vulnerable situations, and the prevalence of comorbidities, as these factors can determine more severe conditions of SARS-CoV-2 infection. Added to this scenario are the effects of the coping measures enacted by each state, which are directly related to the incidence of cases^25^ .

The inequalities in public expenditure for clinical treatment of COVID-19 observed in this study refer to the different scenarios, from the care and epidemiological standpoints, faced by the Brazilian regions and that constitute historical differences in the capacity and coverage of the health system. In this study, the Southeast region had the highest number of hospitalizations and highest total expenditure of hospitalizations for treatment of coronavirus infection. Possible explanations are associated with the higher numbers of infected people concentrated in this region and the greater availability of beds^11^ . Nevertheless, in a country marked by inequalities, including in the provision of health services, Noronha et al.^11^ highlight the presence of care gaps, which can lead local systems to collapse.

The analysis of lethality among COVID-19 inpatients for clinical treatment shows that four of the five states in the Southeast, four of the seven states in the North, and six of the nine states in the Northeast exceeded the average lethality for the country, while the remaining states showed lethality below the national average. The states that registered the higher lethality rate (Paraíba, Alagoas and Roraima) and higher average length of stay (Roraima, Sergipe and Acre) are in the North and Northeast regions. Such data can reveal, to some extent, economic, social and welfare inequalities that, to be solved, require policies that account for regional singularities^25^ .

The availability of resources in certain regions, for example, of dialysis machines and non-invasive and invasive ventilation equipment, may be related to the increased cost of hospitalizations in the others. A detailed mapping of the number of ICU beds and of ventilators and respirators showed that Brazil has a ratio of 15.6 ICU beds per 100,000 inhabitants, with a 7.1 average in the SUS and enormous regional heterogeneity, besides the scarcity of resources in most locations^20^ . These findings corroborate analyses^26 , 27^ conducted by Brazilian researchers regarding the relationship between higher levels of vulnerability to the pandemic and a combination of ICU bed infrastructure below the minimum, mortality from COVID-19-like conditions above the national median^20^ , and increased effects of the disease in poorer regions, such as the North and Northeast^28^ .

In the Brazilian health system, the relationship between public and private becomes even more complex when analyzing the State’s participation in financing supplementary healthcare, regarding the percentage borne by SUS to non-profit and corporate hospitals in the Southern region. It becomes thus urgent to expand the availability of beds by SUS with equitable availability, so that certain areas are prioritized by government investment before the emergency situation established by the COVID-19 pandemic^29^ .

Among the findings presented here, two are most significant. First, the significant impact that COVID-19, in terms of public health expenditures, represents for the burden of hospitalizations for acute respiratory diseases in the country, given the increase in total expenditure on hospitalizations in the analyzed period. Second, and perhaps more important, are the disparities in inpatient expenditure for similar procedures between regions of the country. That said, it is important to analyze the gaps in the process of standardizing clinical and care practices and to elucidate the difficulties and differences found in each location that justify such disparities. Moreover, we must understand which strategies, in the medium and long term, can be implemented to reduce the differences in access, use, and distribution of resources, aiming to lessen the financial impact on the public health system’s accounts.

Some limitations of this study must be emphasized. First, the fact that the data from the last six months available from SIH-SUS are subject to update; but this is the only public domain hospital information system in the country^19^ . The second concerns the evaluation of the system’s coverage, which covers only hospitalizations carried out in the public network and that associated with SUS, excluding hospitalizations conducted privately and by health plans^19^ . Nevertheless, we consider it an important system for analyzing the morbidity profile, since 80% of hospitalizations in Brazil are covered by SIH-SUS, varying according to the level of complexity and procedure required^19^ . Its third important limitation encompasses the absence of an approach on the profile of hospitalized users, considering gender, race/color, age, associated comorbidities, among other aspects that could help evaluating the SUS hospital network during the pandemic. The non-performance of these analyses is due to the unavailability of such variables by SIH-SUS, when working with consolidated IHA data.

The available data do not allow a more detailed approach on the characteristics of users hospitalized for coronavirus treatment. Moreover, the statistics related to hospital morbidity present selective restrictions, providing information only on users whose clinical picture required hospitalization, and partial restrictions, such as users who would need hospitalization but, for some reason, were not admitted^19 , 30^ .

## CONCLUSIONS

The data presented here refer to a specific time frame, which allows us to evaluate the onset of the pandemic in Brazil. Our findings show that the public expenditure represented by hospitalizations for coronavirus treatment, between February and December 2020, cost more than R$ 2.2 billion. Moreover, the results presented here allow us to infer that hospitalizations for this purpose were more costly when compared with those for treating ARI and pneumonia or influenza.

To our knowledge, this is the first study to estimate the cost of SUS hospitalizations for COVID-19 treatment, and may add necessary reflections before the impending collapse of the health care system in most of the country. New cost analysis studies that consider other approaches are needed to monitor the long-term economic impact of the pandemic on the national health system, in addition to the organization and provision of responses that consider local, social, and demographic characteristics. Given this scenario and the role played by SUS in ensuring the lives of thousands of Brazilians during the pandemic, health cannot be seen as an expense, but rather as an investment in defense of the lives of the Brazilian people.
