# Supplementary material for: Public expenditure on hospitalizations for COVID-19 treatment in 2020, in Brazil
Source: Rev Saude Publica. 2021 Aug 4;55:52. doi: 10.11606/s1518-8787.2021055003666 (PMC8352564; doi:10.11606/s1518-8787.2021055003666)
Supplement: Translation

# Translation: Gastos públicos com internações hospitalares para tratamento da covid-19 no Brasil em 2020

**Authors:** Hebert Luan Pereira Campos dos Santos, Fernanda Beatriz Melo Maciel, Geovani Moreno Santos, Poliana Cardoso Martins, Nília Maria de Brito Lima Prado

**Affiliations:** I Brasil Universidade Federal da Bahia. Instituto Multidisciplinar em Saúde - Campus Anísio Teixeira. Vitória da Conquista, BA, Brasil; II Brasil Universidade Federal da Bahia. Instituto Multidisciplinar em Saúde - Campus Anísio Teixeira. Programa de Pós-Graduação em Saúde Coletiva. Vitória da Conquista, BA, Brasil

**Keywords:** Infecções por Coronavirus, Hospitalização, Custos Hospitalares, Gastos Públicos com Saúde, Sistema Único de Saúde

## Abstract

**OBJETIVO:**

Descrever os gastos decorrentes das internações para tratamento clínico de usuários diagnosticados com Covid-19 no Sistema Único de Saúde (SUS) entre fevereiro e dezembro de 2020.

**MÉTODOS:**

Estudo descritivo, elaborado a partir de dados do Sistema de Informações Hospitalares sobre gastos governamentais com internações hospitalares para tratamento clínico de usuários diagnosticados com Covid-19 e das causas incluídas nos capítulos do CID-10. Obteve-se o número de internações, tempo médio de permanência, taxa de letalidade e os gastos totais considerando serviços hospitalares, serviços profissionais e gasto médio por internação.

**RESULTADOS:**

No período avaliado, ocorreram 462.149 internações hospitalares no SUS, sendo 4,9% delas para tratamento de usuários com coronavírus. O gasto total foi superior a 2,2 bilhões de reais, sendo 85% destinados a serviços hospitalares e 15% a serviços profissionais. Os gastos para o tratamento da covid-19 se distribuíram de forma distinta entre as regiões do país. A região Sudeste teve o maior número de internações, maior valor total gasto, maior média de permanência em dias e maior taxa de letalidade, enquanto a região Sul registrou maior porcentagem de gastos com hospitais sem fins lucrativos (58%) e hospitais empresariais (15%).

**CONCLUSÕES:**

As internações para tratamento clínico da infecção pelo coronavírus foram mais onerosas se comparadas às internações para tratamento de insuficiências respiratórias agudas e pneumonias ou influenza. Os resultados demonstraram as disparidades frente aos gastos de internação para procedimentos similares entre as regiões do país, evidenciando a vulnerabilidade e a necessidade de estratégias para diminuir as diferenças no acesso, uso e distribuição de recursos do SUS, garantindo equanimidade, e considerando as injustas desigualdades entre as regiões do país.

## INTRODUÇÃO

A infecção pelo SARS-CoV-2 vem se apresentando como um dos maiores desafios para os sistemas de saúde. Desde seu surgimento, em dezembro de 2019 em Wuhan, na China, a doença causada pelo novo coronavírus já somava mais de 161.513.458 casos confirmados e 3.352.109 mortes em todo o mundo, até 15 de maio de 2021^1^ . Clinicamente, a infecção pode se manifestar de três formas distintas, que variam de acordo a faixa etária e condições clínicas associadas: portador assintomático, indivíduo com doença respiratória aguda (DRA) ou usuários com pneumonia em diferentes graus de gravidade^2^ .

A covid-19 compõe o grupo das infecções respiratórias agudas (IRA), síndromes clínicas cujos agentes infecciosos mais comuns são os vírus respiratórios, como o sincicial, ou bactérias, como *Streptococcus pneumoniae* e *Haemophilus influenzae* ^3^ . A gravidade da doença vai depender do patógeno causador da infecção, do meio ambiente e de fatores do hospedeiro. Dessa forma, de acordo com a Organização Mundial da Saúde (OMS), as IRA estão entre as doenças infecciosas com os maiores índices de morbimortalidade em todo o mundo^4^ . Os dados apontam que essas infecções são responsáveis por mais de 4 milhões de mortes por ano, representando uma das principais causas de óbito nos países em desenvolvimento^5^ .

No Brasil, em períodos anteriores à pandemia, tais infecções chegaram a ser responsáveis por cerca de um quinto das internações hospitalares do Sistema Único de Saúde (SUS)^6^ . Só a Covid-19, até 15 de maio de 2021, registrou mais de 432 mil mortes e 15.519.525 infectados, mas seus impactos no setor saúde e na economia nacional começaram a ser percebidos ainda no início de 2020^7 , 8^ . Cabe destacar que o Brasil é um dos poucos países das Américas a contar com um sistema de saúde universal e gratuito, do qual cerca de 75% da população depende exclusivamente, e que é reconhecido como uma das políticas públicas mais importantes do país^9^ .

O SUS é citado como exemplo de sistema de saúde bem-sucedido na América Latina, principalmente devido à garantia de saúde como um direito de todos os cidadãos brasileiros^9^ . Por outro lado, os desafios do subfinanciamento têm colocado em risco a garantia desse direito^10^ . No contexto da pandemia, o papel desempenhado pelo SUS é notório, especialmente, na atenção hospitalar. A Covid-19 tem demandado taxas adicionalmente altas de hospitalização^11^ , e para além da atenção à pressão gerada sobre o sistema de saúde, cabe observar e analisar os gastos representados para o SUS e como são distribuídos pelo território.

No cenário da pandemia da Covid-19, o desenvolvimento de análises que permitam estimar os custos diretos médico-hospitalares demandados, envolvendo exames, procedimentos, medicamentos, recursos humanos, consultas, internações, reabilitação e outros^12^ , torna-se indispensável para subsidiar a tomada de decisão, no que concerne ao atendimento das demandas nos serviços e ao desenvolvimento de estratégias operacionais pelos sistemas nacionais de saúde^13^ .

Em pesquisa na Biblioteca Virtual em Saúde (BVS), Scientific Electronic Library Online (SciELO) e na PubMed, utilizando os Descritores em Ciências da Saúde (DEcS/Mesh), nos idiomas inglês e português, “custos hospitalares”, “gastos em saúde”, “gastos públicos com saúde”, “despesas públicas” e “gastos públicos com serviços privados” associado aos termos “covid-19” e “Brazil”, através do operador boleano AND, não foram encontrados estudos anteriores a este, de abrangência nacional, que descrevessem os custos para o SUS decorrentes das internações hospitalares para tratamento clínico de usuários confirmados para covid-19. Depreende-se que pesquisas referentes a esses gastos ainda são incipientes na literatura, embora a abordagem dessa temática seja de extrema relevância para compreender os custos ao sistema público de saúde.

Dessa forma, o objetivo do presente estudo foi analisar as proporções dos gastos públicos com internações para tratamento clínico de usuários internados com xcovid-19 pelo SUS no Brasil, entre os meses de fevereiro e dezembro de 2020. Para isso, foram descritos e analisados os gastos públicos com as internações hospitalares e como estão distribuídos em relação às características da população, podendo colaborar para a adoção de medidas capazes de evitar complicações e, consequentemente, o colapso do sistema de saúde no cenário da pandemia.

## MÉTODOS

Trata de um estudo descritivo, a partir de dados secundários sobre gastos governamentais com internações hospitalares para tratamento clínico de usuários diagnosticados com covid-19 no Brasil no período de fevereiro a dezembro de 2020.

Os dados sobre as internações e seus respectivos custos foram coletados a partir do Sistema de Informações Hospitalares do Sistema Único de Saúde (SIH-SUS), disponibilizado no site do Departamento de Informática do SUS (Datasus)^14^ , mediante informações consolidadas das Autorizações de Internação Hospitalar (AIH), classificados por região do país.

As internações selecionadas tinham como procedimento principal o código 03.03.01.022-3 (tratamento de infecção pelo coronavírus) – que corresponde às ações necessárias para o tratamento clínico do usuário internado com diagnóstico de infecção pelo coronavírus, conforme a Portaria nº 245, de 24 de março de 2020, e de acordo com as Orientações técnicas para operacionalização do SIH-SUS durante o estado de emergência de saúde pública por coronavírus, atualizadas pelo Ministério da Saúde em 13 de Agosto de 2020^15^ –, nas unidades hospitalares vinculadas ao SUS (públicas e privadas conveniadas).

Paralelamente, buscou-se comparar os gastos com internações hospitalares para tratamento de paciente diagnosticado com Covid-19, gastos com internações por todas as causas considerando os capítulos do CID-10 e gastos com tratamento das principais infecções do sistema respiratório, tais quais pneumonias, gripe e infecções agudas das vias áreas superiores e inferiores. Para essas, selecionou-se os procedimentos com código: 03.03.14.015-1 – tratamento de pneumonias ou influenza (gripe), 03.03.14.010-0 – tratamento de infecções agudas das vias aéreas superiores e 03.03.14.014-3 – tratamento de outras infecções agudas das vias aéreas inferiores^16^ .

O estudo não considerou a utilização de tratamentos sem comprovação científica para infecção pelo coronavírus. A internação para o tratamento compreende ações e procedimentos necessários para estabilizar e evitar a piora do quadro clínico do usuário. Porém, tais intervenções podem variar substancialmente a depender do quadro clínico de cada usuário (considerando as comorbidades associadas e grau de comprometimento pulmonar), entre os protocolos clínicos adotados por cada estado e município e entre as práticas clínicas assumidas.

O recorte temporal escolhido compreende os meses de fevereiro a dezembro de 2020 para as internações para tratamento clínico de usuários diagnosticados com coronavírus. A delimitação desse horizonte temporal justifica-se, pois em 26 de fevereiro de 2020 foi confirmado o primeiro caso da infecção pelo SarS-CoV-2 no Brasil, e por dezembro constituir o último mês em que foram disponibilizados os dados no SIH-SUS.

As variáveis de interesse do estudo retiradas do SIH-SUS compreenderam: número de internações, valor total das internações, valor dos serviços profissionais, valor dos serviços hospitalares, valor médio de internação (valor médio com internação hospitalar no SUS, por especialidade ou procedimento, em determinado espaço geográfico, no ano considerado), média de permanência (média do total de dias de internação referentes às AIH aprovadas no período) e taxa de letalidade (razão entre a quantidade de óbitos e o número de AIH aprovadas, computadas como internações, no período, multiplicada por 100)^17^ . Para os gastos, considerou-se os valores em reais, e a média de permanência foi contabilizada em dias.

Além disso, buscou-se identificar os valores totais pagos por esfera jurídica, ou seja, a distribuição dos gastos totais governamentais de acordo com a esfera jurídica, considerando as categorias do Cadastro Nacional de Estabelecimentos de Saúde^18^ : “hospitais de administração pública”, “hospitais empresariais” e “hospitais sem fins lucrativos”. O primeiro grupo agrega aqueles que têm como esfera jurídica “administração pública federal”, “estadual ou Distrito Federal”, “municipal” e “administração pública – outros”. Os empresariais são aqueles que têm como esfera jurídica “empresa pública ou sociedade de economia mista” e “demais entidades empresariais”. Já os hospitais sem fins lucrativos são os cadastrados com a esfera jurídica “entidades sem fins lucrativos”^18^ .

Para as tabulações de número, proporção, razão e valores pagos pelas internações, oriundos do SIH-SUS, utilizou-se o programa Microsoft Excel e procedeu-se análise estatística descritiva.

A análise foi realizada sobre dados secundários, de acesso público, sem possibilidade de identificação individual das informações. Assim, consoante as recomendações da Resolução do Conselho Nacional de Saúde (CNS) nº 466, de 12 de dezembro de 2012, foram respeitados os princípios éticos de pesquisas que envolvem seres humanos, sendo dispensada a aprovação do comitê de ética em pesquisa.

## RESULTADOS

Entre fevereiro e dezembro de 2020, foram registrados no SIH-SUS 462.149 internações hospitalares que tiveram como procedimento principal tratamento de usuários com coronavírus, correspondendo a um gasto total de R$ 2.248.011.968,40. Cabe considerar que 85% desse valor foi gasto com serviços hospitalares e 15% com serviços profissionais. Dentre todas as regiões do país, verificou-se que a maior parcela dos gastos ocorreu na região Sudeste, representando cerca de 45% do total. As regiões Sul e região Norte apresentaram, respectivamente, o maior e o menor valor médio por internação ( Tabela 1 ).

**Tabela 1:** Gastos (em reais) das internações hospitalares para tratamento clínico da infecção pelo coronavírusa segundo região do país, Brasil, fevereiro – dezembro de 2020.

Região	Valor total do gasto^b^(R$)	Valor dos serviços hospitalares^c^(R$)	Valor dos serviços profissionais^d^(R$)	Valor médio por internação^e^(R$)
Norte	133.723.986,43	111.708.340,36	22.015.646,07	3.157,96
Nordeste	550.997.791,12	465.375.038,13	85.622.752,99	4.489,91
Sudeste	1.010.529.436,45	857.747.748,55	152.776.809,87	5.154,34
Sul	381.184.490,20	324.887.362,21	56.297.127,99	6.165,64
Centro-Oeste	171.576.264,24	145.114.948,61	26.460.690,51	4.376,16

**Total**	**2.248.011.968,44**	**1.904.833.437,86**	**343.173.027,43**	**4.864,26**

Fonte: Elaboração própria a partir dos dados do SIH-SUS.

^a^Internações hospitalares de pacientes com covid-19 registradas com o procedimento código 03.03.01.022-3, conforme orientação da Portaria nº 245, de 24 de março de 2020 e Orientações técnicas para operacionalização do SIH-SUS durante o estado de emergência de saúde pública por coronavírus publicado em 13 de agosto de 2020 pelo Ministério da Saúde.

^b^Valor referente às AIH aprovadas no período. Este valor não obrigatoriamente corresponde ao valor repassado ao estabelecimento, pois, dependendo da situação das unidades, estes recebem recursos orçamentários ou pode haver retenções e pagamentos de incentivos, não aqui apresentados. Portanto, este valor deve ser considerado como o valor aprovado da produção.

^c^Valor dos serviços hospitalares referente às AIH aprovadas no período.

^d^Valor dos serviços profissionais referente às AIH aprovadas no período.

^e^Valor médio com internação hospitalar no Sistema Único de Saúde (SUS), por especialidade, em determinado espaço geográfico, no período considerado.

Ao considerar todas as internações ocorridas no Brasil no âmbito do SUS, no período analisado, 4,9% tiveram como procedimento principal o tratamento de usuários com coronavírus, correspondendo a 15% dos gastos com todas as internações no país. Ao comparar a média de permanência em dias entre todas as internações ocorridas e as internações ocorridas para tratamento do coronavírus, verificou-se que as internações para tratamento do coronavírus duraram mais tempo ( Tabela 2 ).

**Tabela 2:** Gastos (em R$) e média de permanência (em dias) das internações hospitalares para Capítulos CID-10 X internações para tratamento clínico da infecção pelo coronavírus segundo região do país, Brasil, fevereiro – dezembro de 2020.

Região	Internações para Capítulos CID-10^a^				Internações para tratamento da infecção pelo coronavírus^b^					
	
	Internações	Valor total do gasto^c^(R$)	Valor médio por internação^d^	Média de permanência (em dias)^f^	Internações	%^e^do total de internações	Valor total do gasto^c^(R$)	%^e^do total de valores gastos com internações	Valor médio por internação^d^	Média de permanência (em dias)^f^
Norte	758.143	819.819.970,59	1.081,35	4,7	42.345	5,6	133.723.986,4	16,3	3.157,96	7,4
Nordeste	2.461.730	3.462.075.135,95	1.406,36	5,3	122.719	5,0	550.997.791,1	15,9	4.489,91	7,8
Sudeste	3.772.966	6.511.672.543,21	1.725,88	5,9	196.054	5,3	1.010.529.436	15,5	5.154,34	8,6
Sul	1.646.796	3.055.201.666,37	1.855,24	5,2	61.824	3,7	381.184.490,2	12,5	6.165,64	8,5
Centro-Oeste	746.075	1.064.013.109,82	1.426,15	5,1	39.207	5,2	171.576.264,2	16,1	4.376,16	7,4

**Total**	**9.385.710**	**14.912.782.425,94**	**1.588,88**	**5,4**	**462.149**	**4,9**	**2.248.011.968,44**	**15**	**4.864,26**	**8,2**

Fonte: Elaboração própria a partir dos dados do SIH-SUS.

^a^Todas as internações ocorridas no âmbito do SUS considerando todos os capítulos do CID-10.

^b^Todas as internações ocorridas no âmbito do SUS com o procedimento código 03.03.01.022-3, conforme orientação da Portaria nº 245, de 24 de março de 2020 e Orientações técnicas para operacionalização do SIH-SUS durante o estado de emergência de saúde pública por coronavírus publicado em 13 de agosto de 2020 pelo Ministério da Saúde.

^c^Valor referente às AIH aprovadas no período. Este valor não obrigatoriamente corresponde ao valor repassado ao estabelecimento, pois, dependendo da situação das unidades, estes recebem recursos orçamentários ou pode haver retenções e pagamentos de incentivos, não aqui apresentados. Portanto, este valor deve ser considerado como o valor aprovado da produção.

^d^Valor médio com internação hospitalar no Sistema Único de Saúde (SUS), por especialidade, em determinado espaço geográfico, no período considerado.

^e^As porcentagens foram calculadas tomando como base os valores das internações e gastos totais por região para todos os capítulos do CID-10.

^f^Média de permanência das internações referentes às AIH aprovadas, computadas como internações, no período.

Comparando os gastos e as internações que tiveram como procedimento principal tratamento de infecções agudas, de vias aéreas superiores e inferiores, ou de pneumonia ou influenza, com internações para tratamento do coronavírus, os maiores gastos e número de internações também remetem à infecção pelo coronavírus ( Figura 1 ).

**Figura 1:** Internações hospitalares e gastos para tratamento da infecção por coronavírusa e para tratamento de infecções agudas de vias aéreas inferiores e superiores, pneumonia ou influenzab por região do país, Brasil, fevereiro - dezembro de 2020.

^a^Procedimento com código 03.03.01.022-3 (tratamento de infecção pelo coronavírus) – que corresponde às ações necessárias para o tratamento clínico do paciente internado com diagnóstico de covid-19.

^b^Procedimentos com códigos 03.03.14.015-1 – tratamento de pneumonias ou influenza (gripe), 03.03.14.010-0 – tratamento de infecções agudas das vias aéreas superiores e 03.03.14.014-3 – tratamento de outras infecções agudas das vias aéreas inferiores.

IRA: infecções respiratórias agudas.

Quanto aos gastos associados à média de permanência e à taxa de letalidade, comparando internações para tratamento da Covid-19 a internações para tratamento de pneumonia ou influenza, a região Sudeste apresentou o maior número de internações, maior valor total gasto, maior média de permanência em dias e maior taxa de letalidade. Os estados de São Paulo e Minas Gerais apresentaram os maiores gastos para tratamento dessas condições. Já o maior tempo de permanência para tratamento, tanto da infecção pelo coronavírus como para tratamento de pneumonia ou influenza, foi identificado no estado de Roraima. Os estados da Paraíba e Rio de Janeiro apresentaram as maiores taxas de letalidade, respectivamente, relacionadas a tratamento do coronavírus e tratamento da influenza ou gripe ( Tabela 3 ).

**Tabela 3:** Gastos (em R$), média de permanência (em dias) e taxa de letalidade (por 100 internados) das internações para tratamento clínico da infecção pelo coronavírus X internações para tratamento de pneumonias ou influenza (gripe) segundo região do país, Brasil, fevereiro – dezembro de 2020.Fonte: Elaboração própria a partir dos dados do SIH-SUS

Regiões	Internações para tratamento da infecção pelo coronavírus^a^							Internações para tratamento de pneumonias ou influenza (gripe)^b^						
	
	Internações		Valor total do gasto^c^		Valor médio por Internação (em reais)^d^	Média de permanência (em dias)^e^	Taxa de letalidade^f^	Internações		Valor total do gasto^c^		Valor médio por Internação (em reais)^d^	Média de permanência (em reais)^e^	Taxa de letalidade^f^
			
	(n)	(%)	(R$)	(%)				(n)	(%)	(R$)	(%)			
**Norte**	**42.345**	**9**	**133.723.986,4**	**6**	**3.157,96**	**7,4**	**20,64**	**34.192**	**10**	**34.865.889,09**	**8**	**1.019,71**	**6,0**	**7,79**
Rondônia	4.550	1	18.287.032,7	1	4.019,13	6,2	15,52	2.969	1	4.256.679,93	1	1.433,71	6,4	9,16
Acre	1.566	0	5.902.502,86	0	3.769,16	9,2	22,03	1.059	0	1.426.106	0	1.346,65	8,0	16,9
Amazonas	12.276	3	38.513.890,99	2	3.137,33	8,0	23,37	5.568	2	5.489.313,14	1	985,87	6,2	10,63
Roraima	1.699	0	6.162.828,14	0	3.627,33	9,7	25,13	1.686	0	2.066.920,29	0	1.225,93	9,4	10,74
Pará	18.509	4	54.212.642,19	2	2.928,99	7,2	19,12	19.326	6	18.065.351,26	4	934,77	5,3	6,12
Amapá	2.418	1	4.898.168,88	0	2.025,71	7,2	24,69	1.562	0	1.313.542,34	0	840,94	7,5	5,76
Tocantins	1.327	0	5.746.920,67	0	4.330,76	5,7	19,29	2.022	1	2.247.976,13	0	1.111,76	6,8	8,31
**Nordeste**	**122.719**	**27**	**550.997791,1**	**25**	**4.489,91**	**7,8**	**21,84**	**83.455**	**24**	**93.769.264,42**	**21**	**1.123,59**	**6,2**	**11,16**
Maranhão	18.685	4	55.102.185,73	2	2.949,01	6,9	19,19	14.013	4	13.795.977,17	3	984,51	5,2	6,93
Piauí	9.868	2	46.716.983,14	2	4.734,19	7,4	17,73	9.005	3	7.060.563,32	2	784,07	4,9	6,36
Ceará	23.122	5	95.454.894,14	4	4.128,31	7,7	22,04	16.054	5	17.545.693,07	4	1.092,92	7,0	13,98
Rio G. do Norte	6.464	1	32.079.037,42	1	4.962,72	7,2	24,09	3.774	1	4.317.075,87	1	1.143,9	7,2	12,75
Paraíba	6.136	1	29.309.295,2	1	4.776,61	8,4	31,83	5.757	2	6.396.642,12	1	1.111,11	6,7	14,33
Pernambuco	29.866	6	133.019.014,1	6	4.453,86	8,1	22,34	9.632	3	1.607.5631,99	4	1.668,98	7,6	12,52
Alagoas	5.338	1	239.012.90,05	1	4.477,57	8,6	25,42	4.548	1	6.343.639,79	1	1.394,82	6,3	10,64
Sergipe	4.080	1	19.007.855,07	1	4.658,79	9,2	22,97	2.194	1	2.708.212,73	1	1.234,37	8,1	19,74
Bahia	19.160	4	116.407.236,3	5	6.075,53	8,2	20,34	18.478	5	19.525.828,36	4	1.056,71	5,7	11,35
**Sudeste**	**196.054**	**42**	**1.010.529.436**	**45**	**5.154,34**	**8,6**	**22,61**	**145.005**	**42**	**206.452.552,1**	**45**	**1.423,76**	**7,2**	**16,57**
Minas Gerais	34.553	7	196.447.903,6	9	5.685,41	8,2	19,58	41.118	12	596.589.07,46	13	1.450,92	6,8	13,05
Espírito Santo	10.839	2	68.060.298,71	3	6.279,2	8,7	21,82	6.671	2	9.073.085,62	2	1.360,08	6,6	10,9
Rio de Janeiro	38.865	8	139.621.866,1	6	3.592,48	8,3	27,9	22.279	6	30.502.415,18	7	1.369,11	8,2	22,77
São Paulo	111.797	24	606.399.368	27	5.424,11	8,8	21,78	74.937	22	10.721.8143,8	23	1.430,78	7,1	17,16
**Sul**	**61.824**	**13**	**381.184.490,2**	**17**	**6.165,64**	**8,5**	**19,25**	**58.222**	**17**	**77.888.586,08**	**17**	**1.337,79**	**6,0**	**13,74**
Paraná	22.702	5	131.733.713,6	6	5.802,74	7,9	19,27	22.580	6	30.765.544,92	7	1.362,51	5,2	10,7
Santa Catarina	14.270	3	95.031.703,47	4	6.659,54	8,2	18	14.135	4	19.970.729,02	4	1.412,86	5,6	13,56
Rio G. do Sul	24.852	5	154.419.073,2	7	6.213,55	9,1	19,95	21.507	6	27.152.312,14	6	1.262,49	7,2	17,04
**Centro-Oeste**	**39.207**	**8**	**171.576.264,2**	**8**	**4.376,16**	**7,4**	**18,37**	**27.445**	**8**	**43.362.970,17**	**10**	**1.580,00**	**6,5**	**11,96**
Mato G. do Sul	4.408	1	21.722.376,9	1	4.927,94	8,1	19,44	6.897	2	9.825.686,84	2	1.424,63	6,4	14,21
Mato Grosso	9.328	2	41.813.431,57	2	4.482,57	7,1	19,83	5.813	2	8.298.784,8	2	1.427,63	6,0	10,87
Goiás	16.100	3	70.691.166,46	3	4.390,76	6,5	18,8	10.795	3	18.489.557,09	4	1.712,79	6,1	12,24
Distrito Federal	9.371	2	37.349.289,31	2	3.985,62	8,9	15,68	3.940	1	6.748.941,44	1	1.712,93	8,4	8,88

**Total**	**462.149**	**100**	**2.248.011.968,44**	**100**	**4.864,26**	**8,2**	**21,42**	**348.319**	**100**	**456.339.261,8**	**100**	**1.310,12**	**6,6**	**13,57**

^a^Internações hospitalares de pacientes com covid-19 registradas com o procedimento código 03.03.01.022-3, conforme orientação da Portaria nº 245, de 24 de março de 2020 e Orientações técnicas para operacionalização do SIH-SUS durante o estado de emergência de saúde pública por coronavírus publicado em 13 de agosto de 2020 pelo Ministério da Saúde.

^b^Internações hospitalares de pacientes com registro na Autorização de Internação Hospitalar (AIH) com o procedimento código 03.03.14.015-1.

^c^Valor referente às AIH aprovadas no período. Este valor não obrigatoriamente corresponde ao valor repassado ao estabelecimento, pois, dependendo da situação das unidades, estes recebem recursos orçamentários ou pode haver retenções e pagamentos de incentivos, não aqui apresentados. Portanto, este valor deve ser considerado como o valor aprovado da produção.

^d^Valor médio com internação hospitalar no Sistema Único de Saúde (SUS), por especialidade, em determinado espaço geográfico, no período considerado.

^e^Média de permanência das internações referentes às AIH aprovadas, computadas como internações, no período.

^f^Razão entre a quantidade de óbitos e o número de AIH aprovadas, computadas como internações, no período, multiplicada por 100.

No que diz respeito à porcentagem de distribuição dos gastos totais das internações para tratamento da infecção pelo coronavírus entre as esferas jurídicas dos hospitais do SUS, verificou maior porcentagem de gastos com hospitais sem fins lucrativos (58%) e hospitais empresariais (15%) no Sul do país ( Figura 2 ).

**Figura 2:** Distribuição dos gastos das internações hospitalares para tratamento clínico da infecção por coronavírus entre as esferas jurídicas dos hospitais do SUS por região do país, Brasil, fevereiro – dezembro de 2020.

Fonte: Elaboração própria a partir dos dados do SIH-SUS.

## DISCUSSÃO

No presente estudo, os resultados evidenciaram que os gastos públicos das internações hospitalares para tratamento da Covid-19 se distribuíram de forma distinta entre as regiões do país.

Considerando o volume de internações para tratamento clínico de usuários infectados pelo coronavírus no período analisado, fica evidente o impacto que tais internações geram no sistema de saúde. Embora as internações pagas pelo SUS não representem a totalidade dos casos que necessitam de internação hospitalar, o caráter de evolução progressiva com o qual se desdobra o quadro clínico do infectado confere grande cobertura de assistência ao SIH-SUS para essas internações.

As variações geográficas em torno do valor médio pago por internação no país, constatadas pelas diferenças significativas nos gastos regionais, independentemente da situação pandêmica da Covid-19, também foram relatadas em outros estudos^20^ . Nota-se que o custo médio da internação é maior para esse tratamento quando comparado ao de todas as causas de internação e, mais especificamente, quando comparado aos casos de tratamento de pneumonias ou influenza. Tal fato permite afirmar que as internações para tratamento clínico da Covid-19 são mais onerosas do que aquelas por outras causas. Cabe considerar que as variações entre o valor médio da internação e a média de permanência decorrem de fatores socioeconômicos, epidemiológicos e demográficos, que determinam o perfil da demanda hospitalar pelo SUS, além de políticas públicas de atenção à saúde^21^ .

Ante esse cenário, somam-se as medidas de austeridade fiscal e a forte restrição de receitas pelo nível federal, que são a principal fonte de recursos para a execução dos serviços no SUS. Ainda que tenha sido regulamentada a transferência de recursos financeiros federais específicos para os estados e municípios custearem ações e serviços públicos relacionadas ao enfrentamento da Covid-19, as mudanças normativas recentes no financiamento das ações, com ausência de discricionariedade na aplicação dos recursos pelos entes federados estaduais e municipais, têm intensificado a desigualdade regional na oferta de ações e serviços pelo SUS, inclusive ambulatoriais e hospitalares. Com a aprovação, em 2016, da Emenda Constitucional nº 95, que estabeleceu um teto de gastos para o Governo Federal, o SUS perdeu quase 22,5 bilhões de reais entre 2018 e 2020^22^ . Nesse aspecto, a pandemia impôs novos desafios ao sistema, em virtude das características de transmissibilidade comunitária da doença^23^ e, como demonstrado pelos dados aqui apresentados, do custo mais elevado das internações.

Sabe-se que a maioria dos usuários com Covid-19 evolui com poucos sintomas ou até mesmo assintomática. No entanto, de acordo com a OMS^24^ , cerca de 14% dos casos identificados desenvolvem doença grave, exigindo internação hospitalar e oxigenoterapia, 5% necessitam de internação em Unidades de Terapia Intensiva (UTI), e boa parte dos internados em UTI demandam suporte ventilatório^24^ .

Para a análise dos dados e tomada de decisões, é importante considerar a distribuição heterogênea, entre as regiões do país, da estrutura etária e mobilidade da população, do percentual de pessoas em situação de vulnerabilidade e de prevalência de comorbidades, pois são fatores que podem determinar quadros mais graves da infecção pelo Sars-CoV-2. Soma-se a esse cenário os efeitos das medidas de enfrentamento aplicadas por cada estado, as quais têm relação direta com a incidência de casos^25^ .

As desigualdades em relação aos gastos públicos para tratamento clínico da Covid-19 observadas neste estudo remetem aos diferentes cenários, do ponto de vista assistencial e epidemiológico, encarados pelas regiões brasileiras e que constituem diferenças históricas na capacidade e cobertura do sistema de saúde. Nesta pesquisa, a região Sudeste apresentou o maior número de internações e o maior gasto total para internações para tratamento da infecção pelo coronavírus. Possíveis explicações estão associadas aos maiores números de infectados concentrados nessa região e a maior disponibilidade de oferta de leitos^11^ . Apesar disso, em um país marcado por desigualdades, inclusive, quanto à oferta de serviços de saúde, Noronha et al.^11^ atentam para a presença de vazios assistenciais, que pode levar sistemas locais ao colapso.

A análise da letalidade entre internados para tratamento clínico da Covid-19 aponta que 4 dos 5 estados do Sudeste, 4 dos 7 estados da região Norte e 6 dos 9 estados do Nordeste ultrapassaram a letalidade média do país, enquanto os demais estados apresentaram letalidade abaixo da média nacional. Aqueles que registraram maior taxa de letalidade (Paraíba, Alagoas e Roraima) e maiores tempos médios de permanência (Roraima, Sergipe e Acre) estão nas regiões Norte e Nordeste. Tais dados podem revelar, em alguma medida, desigualdades econômicas, sociais e assistenciais que, para serem sanadas, demandam políticas que levem em conta as singularidades regionais^25^ .

A disponibilidade de recursos em determinadas regiões, por exemplo, de máquinas para diálise e equipamentos de ventilação não invasiva e invasiva, pode estar relacionada ao aumento dos custos das internações nas demais. Um mapeamento detalhado do número de leitos de UTI e de ventiladores e respiradores mostrou que o Brasil possui uma razão de 15,6 leitos de UTI por 100 mil habitantes, sendo a média no SUS de 7,1 e com enorme heterogeneidade regional, além da escassez de recursos na maioria das localidades^20^ . Tais achados corroboram análises^26 , 27^ realizadas por pesquisadores brasileiros a respeito da relação entre os maiores níveis de vulnerabilidade à pandemia e uma combinação de infraestrutura de leitos de UTI aquém do mínimo, mortalidade por condições similares à covid-19 acima da mediana nacional^20^ e acentuação dos efeitos da doença nas regiões mais pobres, como o Norte e Nordeste^28^ .

No sistema de saúde brasileiro, a relação entre público e privado torna-se ainda mais complexa ao se analisar a participação do Estado no financiamento da saúde suplementar, no que concerne ao percentual pago pelo SUS a hospitais sem fins lucrativos e a hospitais empresariais na região Sul do país. Dessa forma, torna-se urgente ampliar a coordenação da oferta de leitos pelo SUS com a disponibilidade equitativa, para que determinadas áreas sejam priorizadas pelo investimento governamental diante da situação emergencial instaurada pela epidemia da covid-19^29^ .

Entre os achados aqui apresentados, há dois resultados importantes. Em primeiro lugar, o impacto significativo que a covid-19, em termos de gastos públicos em saúde, representa para a carga de internações por doenças agudas do aparelho respiratório no país, haja vista o aumento dos gastos totais com internações no período analisado. Em segundo lugar, e talvez mais importante, as disparidades nos gastos com internação para procedimentos similares entre as regiões do país. Isso posto, cabe analisar as lacunas existentes no processo de padronização das práticas clínicas e assistenciais e elucidar as dificuldades e diferenças encontradas em cada localidade que justifiquem tais disparidades. Junto a isso, faz-se necessário compreender quais estratégias, a médio e longo prazo, são possivelmente implementáveis para diminuir as diferenças no acesso, uso e distribuição de recursos, visando reduzir o impacto financeiro nas contas do sistema público de saúde.

Algumas limitações deste estudo devem ser ressaltadas. A primeira refere-se ao fato de os dados dos últimos seis meses disponíveis pelo SIH-SUS estarem sujeitos a atualização. Não obstante, é o único sistema de informação hospitalar de domínio público do país^19^ . A segunda diz respeito à avaliação da cobertura do sistema, que abrange apenas as internações realizadas na rede pública e conveniada ao SUS, excluindo internações realizadas de forma particular e por planos de saúde^19^ . Ainda assim, considera-se um sistema importante para análise do perfil de morbidade, já que 80% do universo das internações hospitalares no Brasil são cobertas pelo SIH-SUS, variando conforme nível de complexidade e procedimento demandado^19^ . A terceira limitação importante compreende a ausência de abordagem sobre o perfil dos usuários internados, considerando gênero, raça/cor, idade, comorbidades associadas, entre outros aspectos que poderiam auxiliar na avaliação da rede hospitalar do SUS durante a pandemia. A não realização dessas análises se deve à indisponibilidade de tais variáveis pelo SIH-SUS, ao trabalhar com dados consolidados das AIH.

Os dados disponíveis não permitem um recorte mais detalhado sobre as características dos usuários internados para tratamento do coronavírus. Além disso, há também o entendimento de que as estatísticas relacionadas à morbidade hospitalar apresentam restrições seletivas, fornecendo informações apenas dos usuários cujo quadro clínico demandou hospitalização, e restrições parciais, como usuários que precisariam de hospitalização, mas, por algum motivo, não se internaram^19 , 30^ .

## CONCLUSÕES

Os dados aqui apresentados referem-se a um recorte temporal delimitado, que permite avaliar o início da pandemia no Brasil. Identificou-se que os gastos públicos representados pelas internações para tratamento da infecção pelo coronavírus, entre os meses de fevereiro e dezembro de 2020, tiveram custo de mais de 2,2 bilhões de reais. Ademais, os resultados ora apresentados permitem inferir que as internações para tal fim foram mais onerosas se comparadas àquelas para tratamento de IRA e pneumonias ou influenza.

Até onde sabemos, este é o primeiro estudo a estimar os custos das internações para tratamento da Covid-19 no SUS, e pode acrescentar reflexões necessárias ante o cenário de eminente colapso do sistema de saúde em quase todo o país. Novos estudos com análises de custos que considerem outras abordagens são necessários para monitorar o impacto econômico da pandemia sobre o sistema de saúde nacional a longo prazo, além da construção e execução de respostas que considerem características locais, sociais e demográficas. Nesse cenário, frente ao papel desempenhado pelo SUS para assegurar a vida de milhares de brasileiros durante a pandemia, a saúde não pode ser encarada como gasto, mas como investimento em defesa da vida do povo brasileiro.
